# The Medical School Gazettes for March

**Published:** 1920-03-20

**Authors:** 


### THE MEDICAL SCHOOL GAZETTES FOR MARCH.

### St. Bartholomew's Hospital Gazette.

IT is stated that the Peace Commemoration Fund appeal, commenced in October of last year, has resulted in upwards of £100,000 being received to date, whilst in addition the amount received towards the fund for erection of a new Home for Nurses is rather over £71,000.

It is proposed by the Editor that the Memorial in honour of the Bart's men who fell in the Great War, numbering 125, shall take the form of a memorial tablet with the names inscribed, and a scholarship at the Medical School of £150.

### The London Hospital Gazette.

THIS contains an excellent portrait of the house governor, Mr. E. W. Morris. Another feature is a page from the life history of "Goliath," a baby which is exciting some interest in the hospital. He is the offspring of a Jewish patient, and the survivor of twins born at the end of the twenty-sixth week of pregnancy. At birth he weighed 2 lb. 1 oz., and was 13 inches long from tip to toe. Against all expectations "Goliath" has continued to live, but then he appears to have enlisted in his aid the whole skill of the London Hospital. Two hours after birth he was given twenty millimetres of human milk by means of a fountain-pen filler, as his mouth was unable to contain a human nipple. For seven days, unless asleep, he was given the same feed mixed at alternate hours with a millimetre of brandy. At the age of a fortnight he was able to take in his mouth a doll's teat fitted to a tiny bottle, and at the age of three weeks he was able to feed himself. On a progressive feeding system he has reached the age of nine months, and weighs 12 lb. The past and present of "Goliath" are interesting—even fascinating. The *Gazette* announces that "he must return to Hesel Street soon, and ——?"

